# Digital Holography as Computer Vision Position Sensor with an Extended Range of Working Distances

**DOI:** 10.3390/s18072005

**Published:** 2018-06-22

**Authors:** Miguel Asmad Vergara, Maxime Jacquot, Guillaume J. Laurent, Patrick Sandoz

**Affiliations:** 1 FEMTO-ST Institute, Université Bourgogne Franche-Comté, CNRS, 25000 Besançon, France; masmad@pucp.edu.pe (M.A.V.); maxime.jacquot@univ-fcomte.fr (M.J.); guillaume.laurent@ens2m.fr (G.J.L.); 2 Sección Física, Departamento de Ciencias, Pontificia Universidad Católica del Perú, Apartado 1761, Lima, Peru

**Keywords:** position sensor, digital holography, image processing, instrumentation, measurement, range finding, pseudo-periodic patterns

## Abstract

Standard computer vision methods are usually based on powerful contact-less measurement approaches but applications, especially at the micro-scale, are restricted by finite depth-of-field and fixed working distance of imaging devices. Digital holography is a lensless, indirect imaging method recording the optical wave diffracted by the object onto the image sensor. The object is reconstructed numerically by propagating the recorded wavefront backward. The object distance becomes a computation parameter that can be chosen arbitrarily and adjusted to match the object position. No refractive lens is used and usual depth-of-field and working distance limitations are replaced by less restrictive ones tied to the laser-source coherence-length and to the size and resolution of the camera sensor. This paper applies digital holography to artificial visual in-plane position sensing with an extra-large range-to-resolution ratio. The object is made of a pseudoperiodic pattern allowing a subpixel resolution as well as a supra field-of-observation displacement range. We demonstrate an in-plane resolution of 50 nm and 0.002deg. in *X*, *Y* and θ respectively, over a working distance range of more than 15 cm. The allowed workspace extends over $12\times 10\times 150\;{\mathrm{mm}}^{3}$. Digital holography extends the field of application of computer vision by allowing an extra-large range of working distances inaccessible to refractive imaging systems.

## 1. Introduction

Computer vision as a measurement system has grown considerably over the last ten years. At the micro-scale, it is a particularly relevant solution because computer vision is intrinsically contact-less and multi-directional. Many works have been reported using computer vision as a measurement system to sense displacements at different scales, from micro-systems [1] to structural engineering [2,3] and also to control microrobots [4,5,6], to assembly micro-parts [7], to determine position versus the six degrees of freedom [8], to calibrate measurement systems [9] or to measure micro-forces by measuring the deformation of flexible structures [10,11,12,13,14].

Both feature-based and area-based methods have been used at the micro-scale but area-based methods are preferred because they are more accurate and more robust to noises [15]. Area-based methods like least squares can achieve nanometer resolutions using regular 20x microscopes [15,16,17,18]. Nevertheless, the measurement range is limited to the field of view, limiting computer vision position sensing of micro-objects to microscopy. It has been showed that direct phase measurements of a periodic pattern can also reach nanometers resolutions [19,20,21]. Associated to a spatial binary code, the range of the measure can be extended to millimeters, far beyond the field of view [22,23]. These pattern-based methods are able to measure the three degrees of freedom in the plane of the image (XYΘ). They have been applied with success to the monitoring of live cell culture [24] and to microrobot calibration [25].

One major drawback of computer vision at micro-scale is the short depth of field that limits out-of-plane measurements. Several solutions have been proposed such as depth-from-focus imaging and confocal microscopy to reconstruct a topography of the scene. Scanning electron microscopy can also be used to get very large depth of field. Anyway, all these methods require a scanning of the scene that slows down the image acquisition rate. Moreover the working distances of these devices are very short and this reduces considerably the interest of a contact-less measure.

Lens-less imaging systems like digital holography can be used in place of refractive devices to address the focusing issues [26,27,28]. Digital holography offers a means for recording the phase and amplitude of a propagating wave front on a solid-state image sensor [29]. Then, by numerically propagating the recorded wavefront backward or forward at particular distances of interest [30,31], different characteristics can be extracted, typically three-dimensional surface or optical thickness images, but also polarization state and intensity distributions. Several recording and processing schemes have been developed to assess diverse optical characteristics that make digital holography a very powerful method for metrological applications [32,33,34,35,36,37].

In this paper, we propose an original approach that combines digital holography and pattern-based methods. Pattern-based methods provide in-plane measurements with nanometer resolutions and millimeter ranges, whereas digital holography enables large and tunable working distances releasing depth-of-field limitations.

The paper starts with a presentation of digital holography as a lens-less imaging system. Then, in-plane position sensing is introduced. The actual performances of the approach are finally evaluated using a dedicated experimental setup.

## 2. Digital Holography as a Lens-Less Vision System

Holography is well known for allowing volume perception of imaged objects thanks to its unique ability to preserve parallax. Principles remain however mysterious to many people and holograms are often considered as almost magical. Better understanding requires a few key-points to be clarified:

- Holography does not record an image of the object but instead the propagating wavefront diffracted by the object onto the hologram plate.
- Holography uses interferences with a reference laser beam to record the amplitude and phase of the propagating wavefront rather than intensity as in usual imaging methods.
- The 2D distribution of light intensity received by the hologram plate produces, after development, thickness (or surface height) variations that behave as a diffraction grating.
- Then, while illuminated by a laser beam similar to the reference beam used at the recording stage, the hologram diffracts the same wavefront as that diffracted by the object at the recording stage. To an observer’s eye, or another imaging device, the distribution of light received is as if the recorded object was still in place.

Originally, holography is based on the use of photosensitive layers with high definitions down to 10nm over areas larger than $1\;m^{2}$. A huge amount of information is therefore recorded that allows an outstanding quality of restitution. However, the chemical process necessary for development is time consuming and most often requires hologram manipulation. These practical modalities as well as the analog character of holography restrict considerably its use in instrumentation. Digital holography was developed to circumvent these limitations. The following presentation is voluntary limited to the basic principles to focus on unique application abilities of digital holography through the case of position sensing with a numerically highly-tunable working distance. Interested readers will find advanced details in extensive literature (see for instance: [29,31,32,33,34,38,39,40,41,42,43,44]).

### 2.1. Specificities of Digital Holography

Digital holography replaces chemical photosensitive layers by electronically-interfaced solid-state image sensors. Despite the physics being exactly the same, the characteristics of solid-state image sensors induce significant differences. On the one hand, solid-state sensors make holograms numerically available. Then, from the knowledge of the reference beam wavefront, object restitution can be performed numerically by back-propagating the recorded wavefront at any distance of the sensor. This suppresses all processing constraints and provides digital holography with versatile and real-time specifications that are of great interest for instrumentation. On the other hand, hologram definitions allowed by solid-state sensors are much lower than that allowed by photosensitive layers. This results directly from the few millimeter extension of solid-state image sensors and from their pixel size of a few microns. Despite continuous technological improvements, these dimensional limitations set supplementary conditions to the application of digital holography:

- The object size is limited, more or less to the same size as the image sensor itself.
- The maximal angle between the interfering beams must remain sufficiently small to ensure that fringes are sampled with at least two pixels per fringe, when off-axis setup is used.
- At the reconstruction stage, the object definition is limited proportionally to the reconstruction distance. The achievable lateral resolution is given by the speckle grain size: λ·d/h, where λ is the laser wavelength, *d* the distance from the image sensor to the reconstruction plane and *h* the lateral extension of the image sensor, assuming d>>h. In a same way, the axial resolution of restitution is given (d>>h), by the following equation:
  (1) $$\sigma_{axial}=\lambda 4d^{2}/h^{2}.$$

Despite these restrictions, digital holography constitutes a highly-sensitive interferometric method working basically with the recording of a single interferogram. The fully-numerical processing of digital holograms has led to numerous applications in diverse fields of instrumentation.

**Figure 1:**
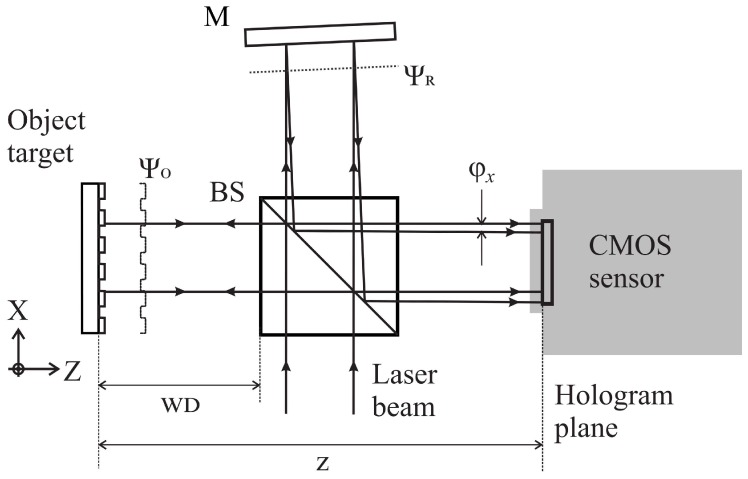
Example of Michelson off-axis digital holography recording setup. BS: Beam splitter cube, M: Mirror, *z*: Axial distance from object to image sensor. WD: Effective working distance.

Figure 1 presents a typical setup used for the recording of off-axis digital holograms. The incident laser beam is split to illuminate both the reference mirror and the object of interest. After reflection, the beam reflected by the mirror and that diffracted by the target are recombined by the beam splitter to interfere onto the image sensor. The reference mirror is slightly tilted to produce a mean angle $\Phi_{x}$ between the interfering wavefronts $\Psi_{0}$ and $\Psi_{R}$. This technique, known as off-axis digital holography, makes the retrieval of the object wavefront easier as explained below.

A key-point here is to notice that, instead of an image of the object, it is the propagating wavefront incident on the image sensor that is recorded. The distance of the object does not impact the recording quality, it only changes the actually recorded wavefront in accordance with light propagation laws. Therefore, blur does not exist at the recording stage of digital holography that is a lens-less imaging system. The object distance stands for a computation parameter that is numerically tunable over an extended range. This specificity makes the range of working distances allowed by digital holography incomparably larger than that allowed by usual refractive imaging devices.

### 2.2. Numerical Object Reconstruction

Digital holography records holograms resulting from the interference of a reference beam with the wavefront diffracted by the object of interest. The recorded intensity distribution can be written as:

(2) $$I\left(x,y\right)={\left|A_{obj}\left(x,y,z=0\right)+A_{ref}\left(x,y,z=0\right)\right|}^{2},$$

where $A_{obj}\left(x,y,z=0\right)$ and $A_{ref}\left(x,y,z=0\right)$ stand, respectively, for the object and reference complex amplitude incident onto the image sensor, *x* and *y* are the lateral coordinates while the distance *z* is counted from the image sensor plane (z=d at the object plane).

**Figure 2:**
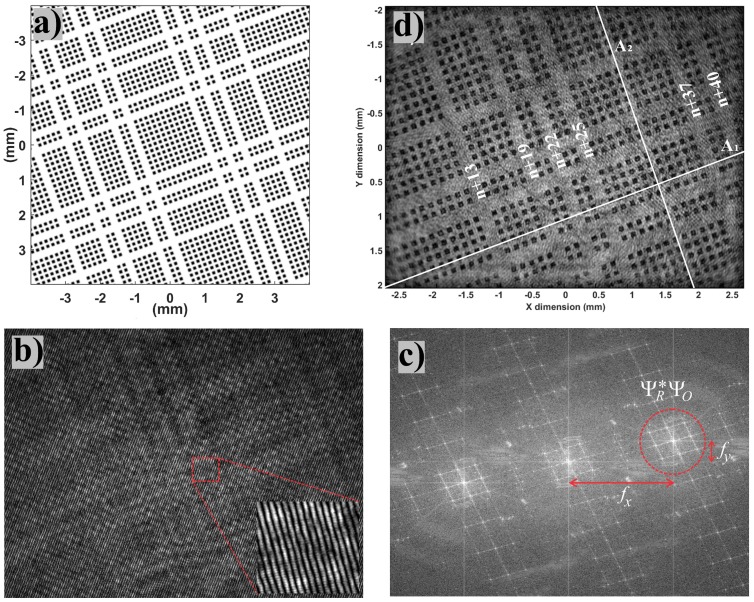
Digital holography process. (**a**) object target, here a pseudo-periodic pattern used for accurate in-plane localization; (**b**) off-axis hologram of the object, the inset shows the interference fringes; (**c**) Fourier spectrum in log scale of the hologram, the discontinuous red circle shows the spatial frequency lobe used for object reconstruction; (**d**) magnitude of object image reconstructed numerically from the hologram (**b**). Data in white are used in Section 3.1.

Figure 2a represents the object target made of an array of square dots and used for in-plane localization by means of digital holography. Figure 2b depicts a typical digital hologram as grabbed by the camera. As shown in the inset, the mirror tilt results in a set of parallel fringes distorted by the irregularities of the wavefront diffracted by the object at a distance *d*. As observed in Figure 2c, the Fourier spectrum of the recorded hologram is made of three separated lobes corresponding to −1, 0 and +1 diffraction orders. Orders −1 and +1 carry complex conjugate information, whereas order 0 is representative for the background intensity. Spectral filtering allows the extraction of the +1 order that is used for further object reconstruction by the angular spectrum of the plane waves approach [30,31,45]. After inverse Fourier transform of the +1 order, the object wavefront is obtained by subtracting the a priori known contribution of the reference wavefront. The latter is provided by the conception of the experimental setup, but supplementary characterization experiments are commonly performed to adjust parameters and compensate for imperfections.

Once the wavefront associated with the object is obtained numerically, it is theoretically possible to compute how it propagates at any distance, either forward or backward, by applying suitable wave propagation laws. This property is responsible for the extended range of working distances allowed by digital holography. The distance between the object and the image sensor is no longer a given characteristic of the imaging device but a computation parameter than can be freely chosen within an extended range. The limits to the working distance are tied to the image sensor dimensions as specified in Section 2.1 provided that the coherence length of the laser source is sufficient. In this aim, object reconstruction consists of back-propagating the object wavefront retrieved from the recorded hologram at a distance *z* from the image sensor that matches the actual distance *d* of the object. Numerically, this can be obtained by multiplying the +1 order by a propagator of plane waves corresponding to the reconstruction distance *z* preliminary to apply the inverse Fourier transform. Such a computation can be repeated for any set of distances *d*, either to find the most appropriate distance value or to build an extended focus image of the object. Figure 2d presents the in-focus image reconstructed by processing the hologram of Figure 2b at an optimal distance of 97.8mm. (Please note that Figure 2a,d do not correspond to the same target zones). More details on object reconstruction algorithms applied to the same target can be found elsewhere [45]. Important issues related to wave propagation algorithms are also discussed in [46], in the frame of a virtual optical propagator system with the use of SLM (Spatial Light Modulator) to restore computed holograms. In our experiment, digital holograms are recorded on a 5 Mp CMOS camera (IDS Imaging Development Systems GmbH, Obersulm, Germany), with pixels of 2.2μm. In this context, as it was widely discussed many years ago [37,47], issues due to sampling effects and limited size of the sensor are taken into account in the propagation algorithms we used, and removed by zero padding and by a hyperGaussian filtering process of the input digital hologram to propagate numerically.

### 2.3. Working Distance and Focus Determination

**Figure 3:**
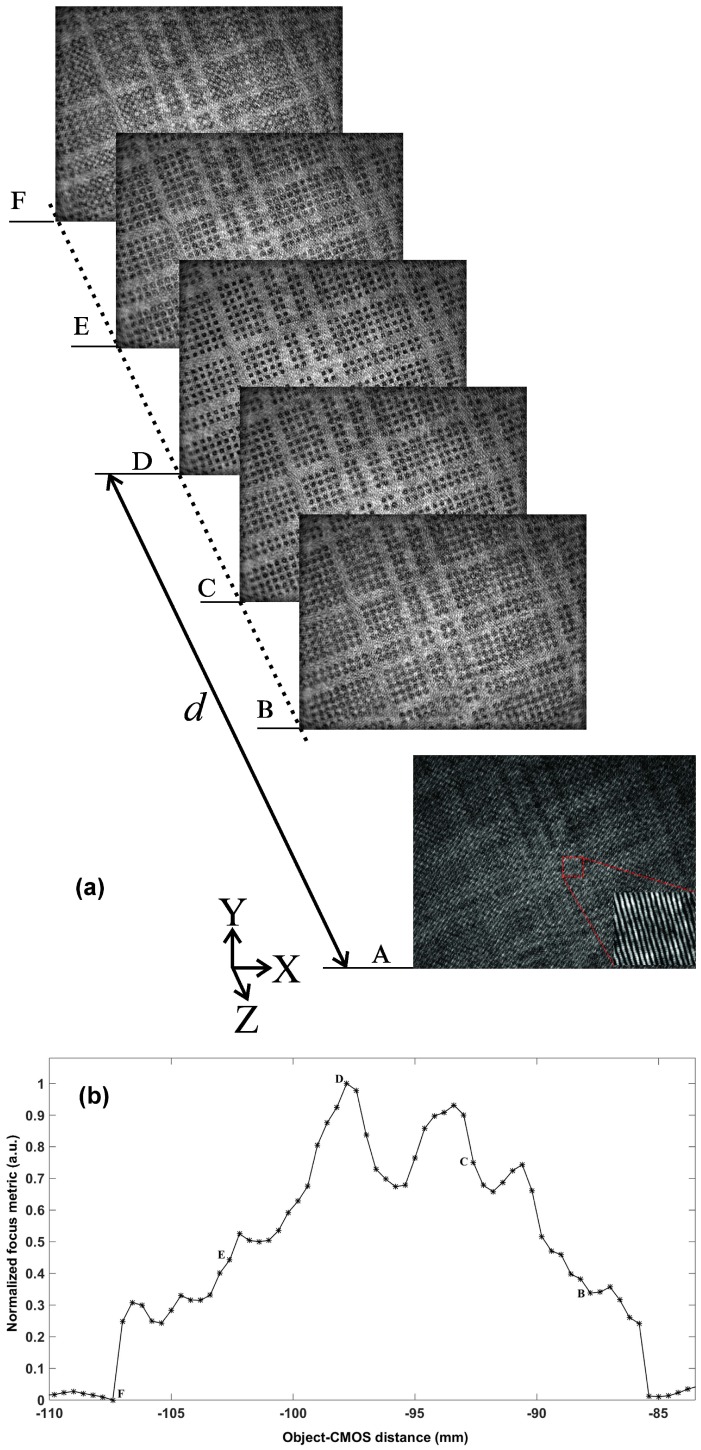
Focus variation: (**a**) object as reconstructed at different distances (B–F) of the image sensor from hologram recorded at distance z=0 (A); (**b**) normalized focus criterion versus reconstruction distance. The curve maximum indicates the reconstruction distance *d* leading to the best in-focus image reconstruction. Letters show the position of reconstructed images B–F. Secondary maximum results from noise in the digital hologram recorded.

The reconstruction distance leading to the best-focused image has to be determined for each position of the object along the *z*-axis. There are various techniques for defining image-formation sharpness-criteria that apply to digital holography [48,49]. The advantage of digital holography is to provide at different restitution distances the complex field diffracted from the z=0 hologram plane. Among the different reconstructed planes (Figure 3a), there is a z=d plane where the image is the sharpest. In our experiments, this working distance can be tuned over an extended range of up to 15 cm. Thus, in the plane of the hologram, the field diffracted by the object is recorded at a given distance, and a first step of numerical reconstruction consists in identifying this distance corresponding to the best focused image reconstruction.

Figure 3a represents five images reconstructed in amplitude, according to different distances *z*, where only image D is in-focus. The sharpness of the image can be determined from multiple focusing criteria such as the sum of the modulus of the complex field amplitude, the use of a logarithmically weighted Fourier spectral function, the variance of gray value distribution, focus measure based on autocorrelation, absolute gradient operator, Laplace filtering or wavelet-based approaches [48,49,50]. In this work, we opted for a criterion based on the spectral weight of the band-pass filtered spatial spectrum of the reconstructed image. This approach is well suited when the angular spectrum of the plane waves method is applied for reconstructing images in digital holography in terms of computational time and accuracy in determining the focal plane. Indeed, for each spectrum of the hologram (Figure 2b), multiplied by the propagator at a given distance *z*, the focus criterion is determined, after a band-pass filtering of the order +1. This consists of summing logarithmically the high frequency spectral components of the filtered spectrum of the restored amplitude distribution. When the focus criterion is at a maximum, the focus of the reconstructed image is optimal. The curve in Figure 3b represents the normalized focus criterion as a function of the reconstruction distance *z* over a range of more than 25mm with a pitch of 1 mm. We observe a main maximum at z=97.8 mm, corresponding to the best-focused reconstructed image and fitting with the actual distance as measured manually. Despite noise remaining significant in the digital hologram recorded and generating secondary peaks on the curve in Figure 3b, the distance obtained from the main peak is correct, and this method would allow an automated determination of the optimal reconstruction distance [28,51,52]. In this example, the precision of the estimate of *d* depends on the axial resolution given by Equation (1), and is about 800μm in this case. However, maximum curve approximation methods can be applied to optimize focus distance detection. It should also be noted that digital holography gives access to the complex amplitude of the diffracted field, and, unlike conventional imaging, it is possible to exploit the real and imaginary part of the reconstructed field distribution to refine the focus criterion. For example, in the case of a pure amplitude object, the imaginary part of the reconstructed image will be zero when the image is in focus.

## 3. Application to In-Plane Position Sensing

### 3.1. Position Sensing

Basically, as a vision system, digital holography is compatible with most visual measurement methods. We choose to emphasize its unique capabilities, especially a highly tunable working distance, through the case of in-plane position sensing. The high-performing approach used in this paper was developed in our laboratory [23,45,53]. The method is based on a pseudo-periodic pattern fixed onto the moving target. The pseudo-periodic pattern is made of a thin aluminum layer deposited onto a glass substrate and micro-structured in a clean room by photolithography. After aluminum etching, we obtain a periodic frame of dark dots (lack of aluminum) contrasting with the bright, reflective, aluminum background as can be observed in Figure 2c. Certain lines and columns are missing, thus distorting the periodic frame in a unique manner. The distribution of missing lines and columns corresponds to an absolute encryption of line and column indexes with respect to the whole pattern that is much larger than the vision-system field-of-observation. Thanks to this absolute position encoding, any partial zone of the pattern observed can be localized in an absolute way within the whole pattern. The encoding is based on linear shift register sequences and makes the allowed range of lateral displacement independent of the field of observation of the vision system used [23,24,45].

The coarse but absolute position determination allowed by the binary encryption of line and column indexes is combined with a high-resolution but relative position determination based on pattern periodicity [23,24,45,53]. The method, based on phase computations, exploits the narrow spatial frequency band associated with the pattern periodicity to filter out noises outside this band as well as to perform data averaging over the entire image. The resulting performances are highly sub-pixellic with a typical lateral resolution of a thousandth of a pixel.

**Figure 4:**
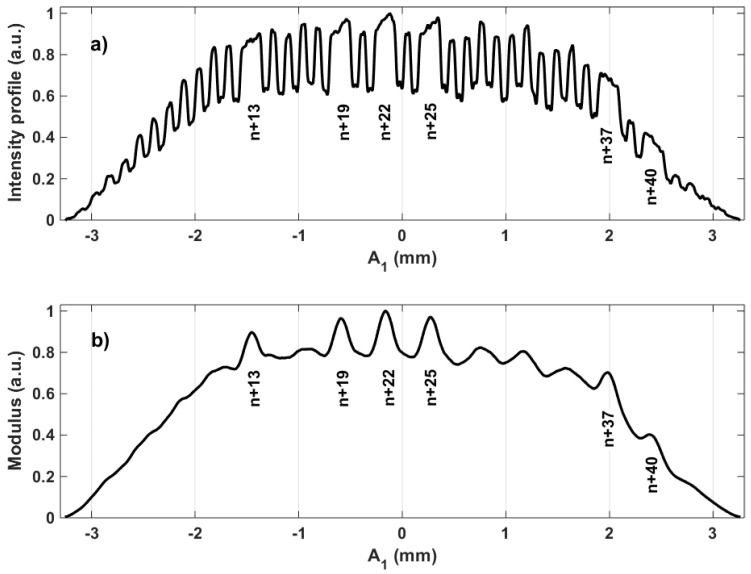
Intermediary signals used for both high-resolution and coarse position detection: (**a**) intensity profile along direction $A_{1}$ of Figure 2c after summation versus direction $A_{2}$. The periodicity leading to high-resolution appears clearly; (**b**) the local magnitude at the pattern frequency allows the localization of the missing lines with respect to pattern direction $A_{1}$.

Figure 4 presents intermediary signals used for both high-resolution and coarse position detection. Figure 4a depicts the intensity profile along pattern direction $A_{1}$ after summation along $A_{2}$ as plotted in Figure 2c. The signal periodicity used for relative but high-resolution measurement is clearly visible. Figure 4b presents complementary information through the local magnitude associated with the pattern frequency that allows the identification of the missing columns necessary for coarse position determination. A similar processing is applied in the perpendicular direction by inverting $A_{1}$ and $A_{2}$ to obtain complementary u,v positions. We note the coordinates x,y when referring to the image pixel frame and u,v when referring to the pattern directions. The angle between the x,y and u,v coordinate systems determines the pattern orientation with respect to the image pixel frame. The latter is also obtained by means of phase computations. Furthermore, to release the π/2 ambiguity affecting this determination of pattern orientation, supplementary line and column indexes are identified in order to detect the direction of increasing distances with respect to the reference corner of the whole pattern. More details on the retrieval of the in-plane orientation can be found elsewhere [23,24,53].

Once reconstructed numerically, the images of the pseudo periodic pattern observed by digital holography are processed to determine their position with respect to the image sensor. Results obtained provide with a high-accuracy the position of the pseudo-periodic pattern that corresponds exactly to the center of the image sensor as well as the pattern orientation with respect to the sensor pixel frame. The a priori knowledge of the period of the pseudo-periodic pattern, 150μm in our case, allows the conversion of line and column indexes into actual positions and displacements without requiring any calibration procedure. Details on image processing involved can be found elsewhere [23,45,54]. They are not described here to save room for emphasizing on performances allowed by digital holography used as a visual-based position sensor.

The ultimate positioning resolution permitted by processing periodic pattern images is discussed theoretically elsewhere on the basis of numeric simulations [55]. This paper provides an abacus determining the resolution that can be expected as a function of the pattern period, the quantization depth of the camera and of the number of periods present in the field of observation. The effect of Gaussian noise on positioning resolution is also discussed. These results were obtained for a vision configuration using white light and a refractive imaging system. In our case, imaging is based on digital holography that involves laser illumination and speckle noise in the numerically reconstructed images rather than white light illumination and Gaussian noise, respectively. Nevertheless, the impact of these differences on the expected resolution is not significant as long as the speckle grain size (λ·d/*h*) remains small in regard to the pattern period as it is the case (less than 5μm versus 150μm). From the abacus provided in this previous paper, an ultimate lateral resolution of about one nanometer can be expected in our digital holography application using a period of 150μm and a camera with a 8 bit quantization. The experimental resolution achieved will be discussed in regard to this theoretical value in Section 4.2.

### 3.2. Experimental Setup and Actuators Used

**Figure 5:**
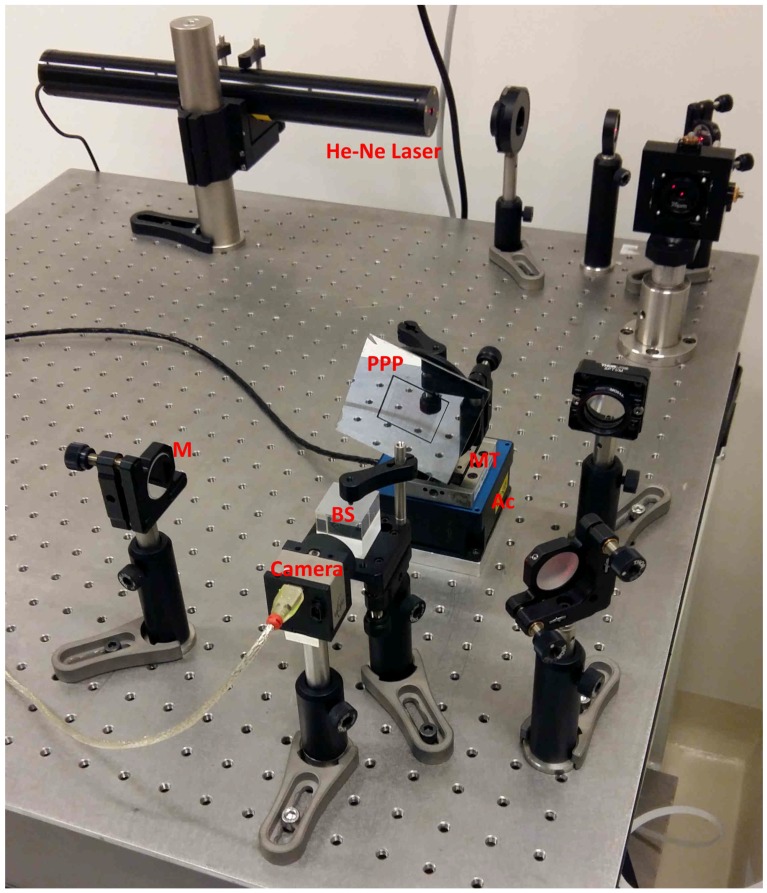
Photograph of the experimental setup. After expansion, a 632.8 nm He-Ne laser beam illuminates the pseudo-periodic pattern (PPP) and the reference mirror (M). Reflected beams interfere onto the camera sensor after recombination by the beam splitter (BS). Ac: servo-controlled actuator, MT: manual axial translator.

The experimental setup used is depicted in the photograph of Figure 5. It is made of a Michelson interferometer illuminated by a 632.8nm He-Ne laser beam. The laser beam is expanded to produce an almost uniform intensity distribution over a diameter of about 1cm. The camera is a μ-eye USB2 UI-1480-SE-M from IDS-Imaging (Obersulm, Germany) with 2560×1920 pixels with $2.2\times 2.2\;\mu m^{2}$ area. The pseudo-periodic pattern used as mobile target presents a periodic frame with a period of 150μm (cf. Figure 2c). The encryption of the absolute position is based on 6-bit words with three lines (resp. columns) per bit. The unambiguous determination of the absolute position requires the decoding of three consecutive positions; i.e., 8 bits or 24 periods. A correct position measurement thus requires the observation of a minimum area of $3.6\times 3.6\;{\mathrm{mm}}^{2}$ that corresponds to 1.6% of the full pattern size of $28\times 28\;{\mathrm{mm}}^{2}$.

During experiments, the pseudo-periodic pattern was successively fixed onto the following servo-controlled actuators from Physik Instrumente (Karlsruhe, Germany):

- M-037.DG rotation motor; range of 360 deg., $2.10^{-4}$ deg. minimum incremental motion and 0.012 deg. backlash.
- Two crossed M-111 micro-translation stages with 15mm range, 50nm ultimate incremental motion, 0.1μm incremental repeatability and 2μm backlash.
- P-615.3CD piezo-controlled nanocube with $350\times 350\times 250\;\mu m^{3}$ *XYZ* range with 1nm resolution used along the *YZ* axis.

The allowed working distance range was explored manually by shifting the *Z*-distance between the pseudo-periodic pattern and the camera by means of a manual translation stage (25mm range) and by fixing the holding part to next mounting holes (1 inch apart). In this way, in-plane position measurement tests were repeated at various *Z*-distances between the pseudo-periodic pattern and the camera. The actual *Z*-distance was measured manually with a millimeter resolution that is acceptable in regard to the depth-of-focus of reconstructed images. The working distance WD corresponds to this *Z*-distance but counted from the distal face of the beam splitter cube that determines the closest target position allowed (in practice, Z−WD=62mm).

At every working distance tested, hologram recordings were synchronized with computer-controlled displacements using Matlab routines (R2014b, MathWorks Inc., Natick, MA, USA). Whenever possible, internal sensors of actuators were read at each position explored to provide reference position values for evaluating measures obtained by digital holography. Recorded holograms were post-processed for reconstructing the corresponding images of the pseudo-periodic target and then for determining the in-plane position associated. Finally, the series of measures obtained in this way are compared with the control inputs applied and positions read from internal sensors to evaluate the performances achieved in position sensing by digital holography.

## 4. Results and Working Distance Range

Two series of experiments were carried out for exploring the method capabilities to retrieve, respectively, in-plane rotations and in-plane displacements. In each case, several working distances were tested by shifting the holding part on the optical table. The range of working distances explored is limited to about 10cm in the case of rotation because of setup configuration. For lateral displacements, we arranged the optical devices in a slightly different way to allow the exploration of more than 15cm of working distance range. Results are presented in the following sub-sections.

### 4.1. In-Plane Rotation

**Figure 6:**
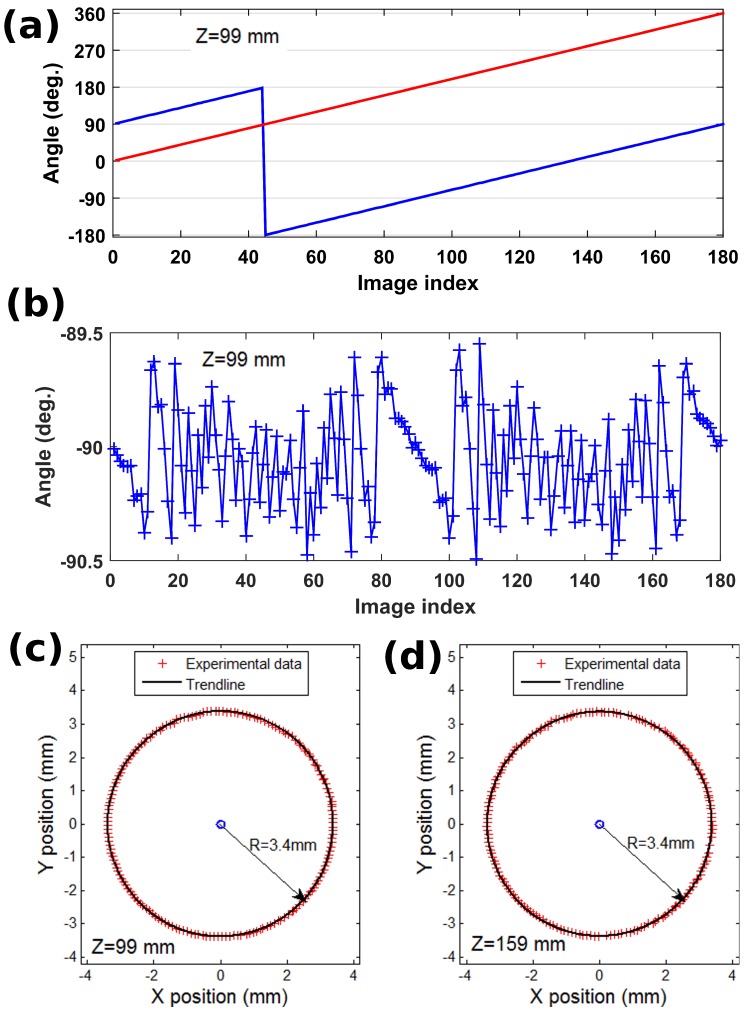
Results from rotation measurements with 2deg. increments. (**a**) reconstructed angle (red) versus angle given by the internal sensor of the actuator (blue) at a distance of 99mm; (**b**) angle deviation from actuator internal sensor after unwrapping; (**c**,**d**) XY displacements produced by rotation with respect to the center pixel of the camera at distances of 99mm and 159mm.

Figure 6a compares the reconstructed in-plane orientations to the reference angles provided by the internal sensor of the rotation actuator at a distance of 99mm. The full 2π range was explored by steps of 2deg. Results show a mean angle mismatch of ∼90deg. that results from the initial orientation of the pseudo periodic pattern on the motor. The 2π jump is due to the combination of the initial orientation of the pattern and from the mismatch between the unambiguous ranges of our detection method ]−π,+π] and of the motor internal sensor [0,360[. This jump is easily unwrapped and we observe the measurement error plotted in Figure 6b by using the motor internal sensor as a reference. The angle deviation presents some random parts and some more systematic errors at specific positions. This was attributed to a positioning error due to backlash induced by successive back and forth adjustments by the servo-control loop that result in cyclic errors. The method works satisfactorily with a linearity of 0.31% and a standard deviation of 0.219deg. at this particular working distance of 99mm. Table 1 summarizes the results obtained in rotation for the diverse working distances tested. It shows that the level of performance decreases very slightly when the working distance increases despite the resolution of the reconstructed images decreases with distance (cf. Equation (1)).

Figure 6c,d present the (x,y) pattern displacements produced by the rotation at two different working distances of 99mm and 159mm. The radius of the circle observed corresponds to the distance of the actual center of pattern rotation from the optical axis defined by the normal of the image sensor at its central pixel. This radius is the same across the different experiments because the optical axis was almost perfectly perpendicular to the lines of mounting holes on which the holding part was successively fixed.

**Figure 7:**
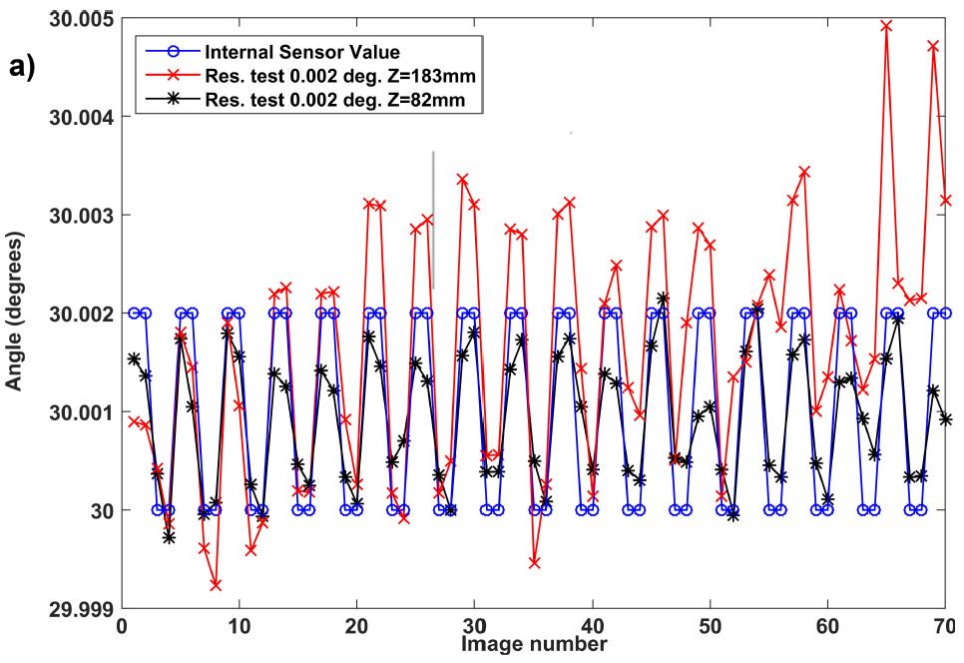

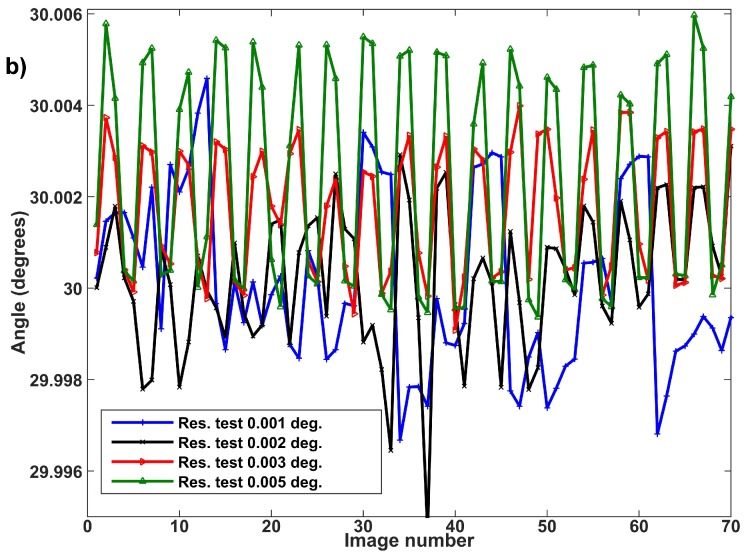
Resolution in rotation observed from measurement tests with: (**a**) steps of 0.002deg. at distances of 82mm and 183mm respectively; (**b**) steps of 0.005, 0.003, 0.002 and 0.001deg. at a distance of 183mm.

The allowed angular resolution was explored by applying a square signal to the actuator with decreasing amplitudes from 0.01deg. to 0.001deg. Figure 7a presents the results obtained with an amplitude of 0.002deg. at extreme working distances explored in rotation; i.e., 82mm and 183mm. The square excitation is clearly visible on the reconstructed signal despite distorted amplitude and slight drifts. Similar results were obtained for the other working distances tested. A resolution of 0.002deg. is therefore demonstrated over a full working distance range of more than 100mm. Figure 7b presents complementary results with the angles retrieved experimentally while square signals of decreasing amplitudes were applied to the rotative actuator at a distance of 183mm. As expected, the visibility of the square shape decreases with the reduction of the signal amplitude. It is perfectly visible for amplitudes of 0.005deg. and 0.003deg., disturbed by noise for an amplitude of 0.002deg., and hardly recognizable for an amplitude of 0.001deg. In all our experiments with an amplitude of 0.001deg., we observe the presence of the excitation square signal in the results obtained but with a level of non-random noise that makes result interpretation ambiguous. This noise is suspected to come from the actuator of which minimum incremental motion is only fivefold smaller and of which servo-control induces back and forth motions resulting in backlash errors. Despite us being convinced that the intrinsic resolution capabilities of the method are below 0.002deg., we claim this resolution that was clearly demonstrated with the devices used.

### 4.2. In-Plane Displacements

**Figure 8:**
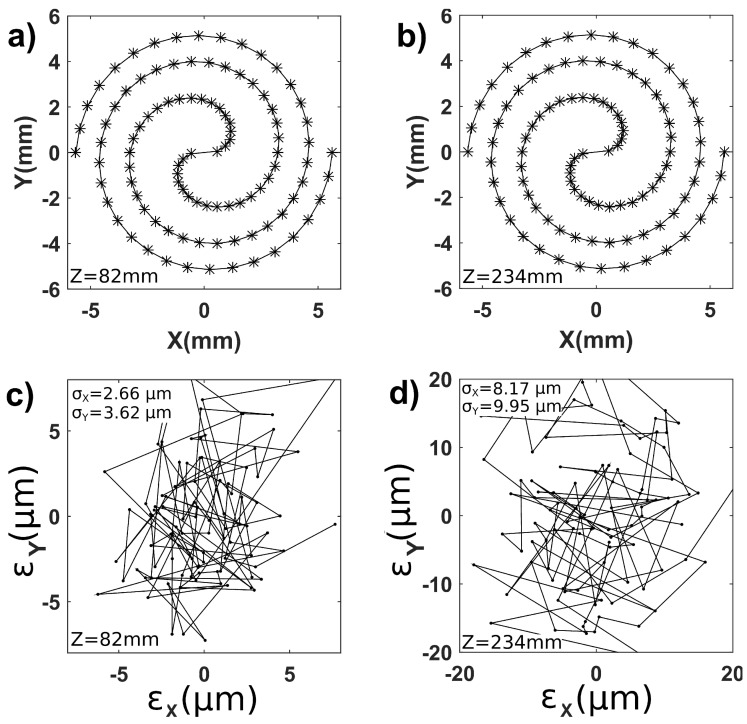
Reconstruction of extended lateral displacements. Reconstructed XY trajectory at (**a**) 82mm and (**b**) 234mm. XY deviations from actuator sensor values at (**c**) 82mm and (**d**) 234mm.

The extended measurement range of the method was explored by applying a kind of spiral trajectory to the crossed linear motors (M-111). Figure 8a,b present the reconstructed trajectories for the extreme working distances of 82mm and 234mm. The trajectories cover an area of about $12\times 10\;{\mathrm{mm}}^{2}$ to be compared with the image sensor size of $5.6\times 4.2\;{\mathrm{mm}}^{2}$ (ratio 5). This extended lateral measurement range is allowed by the binary code encrypted in the pseudo-periodic pattern that makes the allowed displacement independent of the field of view of the camera.

Figure 8c,d present the deviation of the reconstructed (*x*,*y*) positions from the values returned by the internal sensors of the actuators. These deviations are quite large and were attributed to the stack of motors that converts out-of-plane spurious motions into in-plane displacements. For instance, for a motor thickness of 2cm, an out-of-plane rotation of the supporting motor of 0.1deg. induces a 35μm lateral displacement of the external surface of the supported motor.

**Figure 9:**
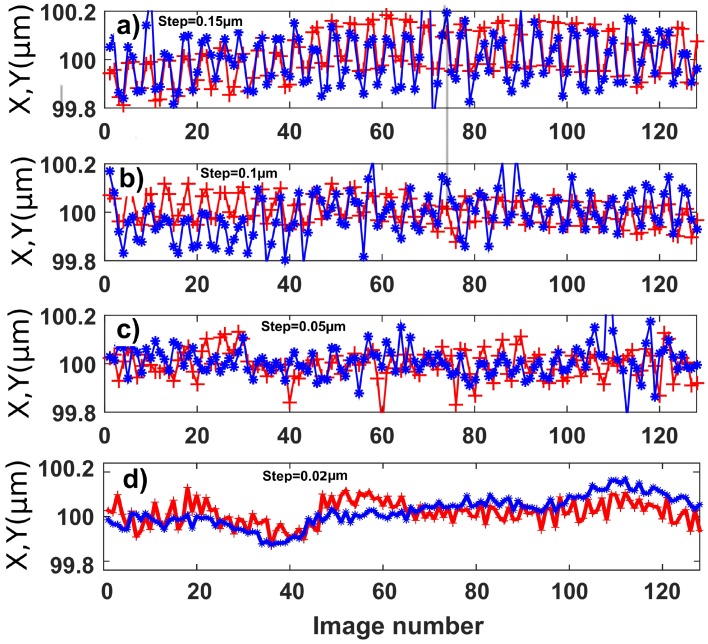

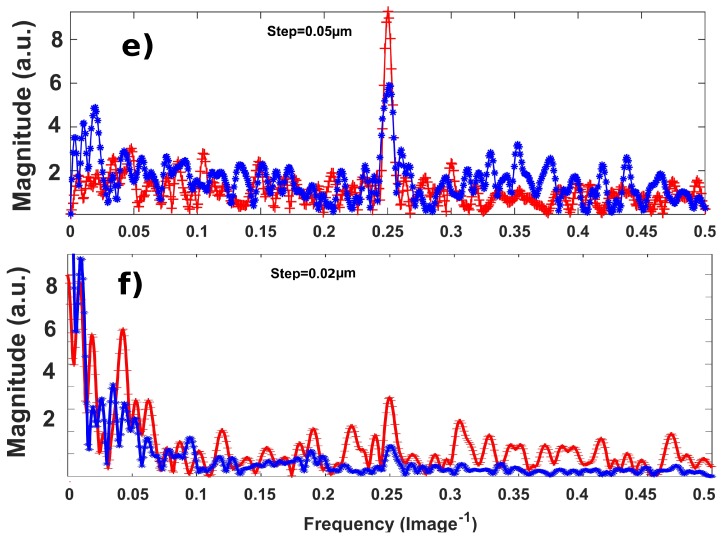
Results of resolution test along *X* (red) and *Y* (blue) directions at a median distance z=151mm. Steps of (**a**) 0.15μm; (**b**) 0.10μm; (**c**) 0.05μm, (**d**) 0.02μm; (**e**,**f**) power spectrum of the data series with steps of (**e**) 0.05μm and (**f**) 0.02μm. The frequency of the input command applied is of $0.25\;{\mathrm{frame}}^{-1}$.

The lateral resolution capabilities of the method were explored with a more accurate actuator (P-615.3CD) and by applying a square signal of decreasing amplitude, successively along the *X* and *Y* directions. Results are reported in Figure 9 and are similar in *X* and *Y* directions. The square signal is clearly visible for amplitudes of 0.15μm (Figure 9a) and 0.10μm (Figure 9b). The square signal is present but less clearly visible for an amplitude of 0.05μm (Figure 9c). However, if we perform a Fourier transform of the reconstructed signal, a peak is observed at frequency 0.25 which corresponds to the excitation signal frequency (Figure 9e). The presence of this spectral lobe confirms that oscillations observed in Figure 9c are related to the excitation signal applied. A supplementary test was performed with an amplitude of 0.02μm (Figure 9d). In this last case, the excitation signal is no longer visible in the reconstructed displacements. In the corresponding Fourier spectrum (Figure 9f), peaks are still detected at the expected frequency but with a magnitude that is no longer distinguishable from background noise. Thanks to this set of experimental results, a lateral resolution of 0.05μm can be claimed. This resolution is 50 times larger than the ultimate resolution discussed theoretically in Section 3.1. Such a ratio between theory and experience is common, especially in the sub-micrometer range where environmental disturbances due to thermal and mechanical drifts are no longer negligible.

No optimization of digital data processing was carried out in our proof-of-principle experiments and it takes about 2s to process each hologram using Matlab. However, the high-speed reconstruction of digital holograms, up to 150fps, was demonstrated by different groups. It is notably the case of the DHM (Digital Holographic Microscope) developed and commercialized by Lyncée Tec SA (CH-1015 Lausanne, Switzerland). Such fast processing tools could be applied to our position sensor to speed up the measurement rate beyond 100fps.

## 5. Conclusions

This paper explores digital holography as a vision method for sensing applications. In comparison with usual refractive imaging systems, digital holography requires a laser light source and an interferometric setup that may be seen as supplementary requirements that are too demanding. We agree that, for usual vision needs, conventional refractive imaging systems are preferable. However, digital holography presents specific features that may extend the field of application of visual methods. We deal in this paper with the property of digital holography to comply with a large range of working distances. Indeed, digital holography is based on the recording of the optical wave incident on the image sensor, whatever the distance of the diffracting object of interest. Therefore, all requirements tied to the use of refractive lenses, especially the working distance, are canceled in digital holography. The only restrictions are tied to the allowed definition of holograms as defined by the speckle grain size and that forms a criterion analogous to the numerical aperture of refractive lenses (cf. Equation (2)).

**Figure 10:**
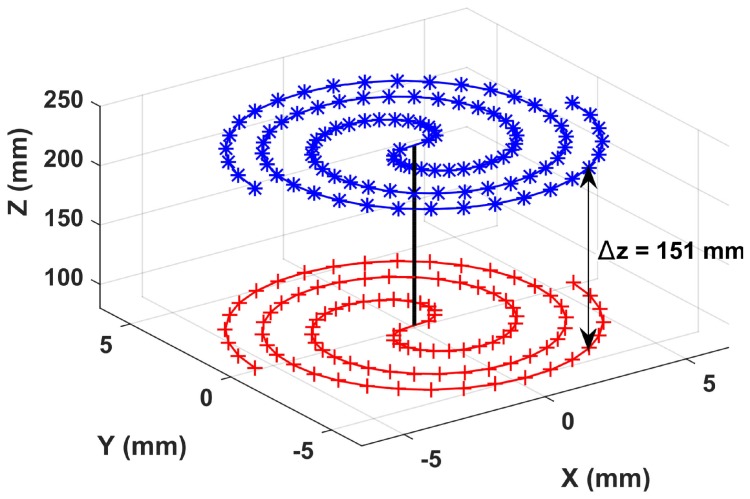
The same in-plane trajectory reconstructed at extreme working distances separated by more than 15cm.

The unique capabilities offered by digital holography are highlighted in Figure 10, which superimposes two in-plane trajectories reconstructed at extreme working distances more than 15 cm apart. The allowed measurement volume is about $12\times 10\times 15\;{\mathrm{mm}}^{3}$ and is compatible with resolutions of 0.05μm and 0.002deg. for in-plane displacement and in-plane orientation, respectively. To the best of our knowledge, no other imaging system would allow the same level of performances. For imaging purposes, resolutions in the nanometer range require some kind of super-resolution methods to overcome the diffraction limit. This is different in position sensing applications where averaging across the observed field provides an additional means to increase the amount of information that results in improved resolutions [19]. The use of pseudo-periodic pattern as in this paper is an efficient way to implement such averaging processing by rejecting noise outside the narrow bandwidth associated with the dot distribution. Combined with digital holography, the method results in unique capabilities allowing lateral resolution in the nanometer range together with a working distance range of more than 15cm. In comparison, usual refractive imaging systems would require a tradeoff between resolution and working distance range. Indeed sub-micrometer resolution requires high magnification objectives, at least 10×, of which working distance is limited to a few millimeters to be compared with 15cm using digital holography.

In this paper, we do not discuss the measurement of the object distance *Z* that could be retrieved numerically with a low resolution (about 1mm) from best focus determination. We only adjusted the correct *Z* distance to perform three degrees of freedom position measurements (in-plane) at diverse working distances within a 15cm range.

Beyond the unique measurement capabilities reported in this paper, two major directions could be explored further. On the one hand, digital holography is an interferometric method that provides phase maps. The latter could be used to address out-of-plane rotations or to follow the z-displacements of the object provided that 2π ambiguities are monitored properly [21]. On the other hand, we processed in this paper in a sequential manner by first reconstructing the object image and second by retrieving position from the patterned image obtained. However, the position information is already present in the digital hologram and one may try to retrieve an in-plane position without reconstructing the object image. This may result in a significant reduction of the computation cost and in releasing the necessity to determine the object distance *Z*.

Finally, digital holography has been explored for a restricted number of applications, notably biological imaging and particle image velocimetry, and remains poorly known in other engineering communities. Hopefully, this paper may draw the attention of computer vision and position sensing communities on the unique capabilities offered by digital holography.

## Figures and Tables

**Table 1:** Data from rotation measurement tests.

Experim. WD (mm)	Experim. Z (mm)	Reconst. Distance (mm)	Standard Deviat. σ (deg.)	Linearity δ/R (%)
20	82	82.5	0.201	0.2
37	99	97.8	0.219	0.3
97	159	156.2	0.267	0.3
121	183	183.0	0.229	0.3
